# RARG Gene Dysregulation in Acute Myeloid Leukemia

**DOI:** 10.3389/fmolb.2019.00114

**Published:** 2019-10-24

**Authors:** Maria Rosa Conserva, Immacolata Redavid, Luisa Anelli, Antonella Zagaria, Giorgina Specchia, Francesco Albano

**Affiliations:** Hematology Section, Department of Emergency and Organ Transplantation (D.E.T.O.), University of Bari, Bari, Italy

**Keywords:** retinoic acid receptor γ, acute promyelocytic leukemia, acute myeloid leukemia, gene fusions, protein fusions

## Abstract

Retinoic acid receptor γ (RARγ) belongs to the nuclear receptor superfamily and shares 90% homology with retinoic acid receptor α (RARα) and retinoic acid receptor β (RARβ). *RARA* rearrangements are well-known to be involved in acute promyelocytic leukemia (APL), but *RARG* rearrangements can also resemble this kind of leukemia. In this review we trace the role of RARγ, considering both its physiological and oncogenic contribution; from 2011 to date, nine cases of patients harboring *RARG* fusions have been reported. These patients showed typical APL features, including the clinical presentation, coagulation abnormalities and morphological features of bone marrow (BM), but are not responsive to APL standard therapy. We stress the urgent need for a better comprehension of the critical role of *RARG* dysregulation in the leukemogenesis process, since optimum therapy strategies have not yet been established.

## Introduction

Acute myeloid leukemia (AML) is a hematologic malignancy that may arise in patients with another hematological disorder already present or after previous therapy, for instance following previous exposure to alkylating agents or DNA topoisomerase inhibitors or radiation (Sill et al., 2011), but most frequently it appears as a de novo malignancy in previously healthy individuals (De Kouchkovsky and Abdul-Hay, 2016). The bases of the pathogenesis reside in an abnormal and malignant clonal expansion of specific progenitor myeloid cells that do not complete their physiological differentiation process (De Kouchkovsky and Abdul-Hay, 2016). The cause is to be found in a genetic alteration of the progenitor hematopoietic cell that gives life to a clonal population of blast cells, with the manifestation of an atypical hematopoiesis (Levine, 2013; Thomas and Majeti, 2017). Already in 1957, APL accounted for 5–15% of the total AML cases (Szotkowski et al., 2015). In fact, APL is a different subtype of AML characterized by an accumulation and expansion of leukemic cells that fail to go beyond the promyelocyte stage of myelopoiesis (de Thé et al., 1991). Before the discovery of all-trans retinoic acid (ATRA) and arsenic trioxide (ATO), patients with APL had a high risk of mortality, due to hemorrhagic complications before and during induction therapy (Choudhry and DeLoughery, 2012). Both ATRA and ATO behave as differentiating agents, since they promote the differentiation and maturation of leukemic promyelocytes to neutrophils (Asou et al., 1998; Karim et al., 2014; Daver et al., 2015). According to the current WHO criteria, the presence of *t*(15;17)(q22;q12), hence of the promyelocytic leukemia protein (*PML)-RARA* gene fusion, allows an AML to be classified as APL, but other variant *RARA* translocations with other partner genes are not only considered distinct, but not all have typical APL features and indeed, some patients show resistance to ATRA (Arber et al., 2016). In particular, in a subset of patients the *PML-RARA* fusion is not detected, but other rearrangements are identified that involve *RARB* and *RARG*, two distinct isoforms of *RARA*, that cause a pathological phenotype that resembles APL (Marinelli et al., 2007; Osumi et al., 2018). The aim of this review is to focus on the various emerging cases of APL without the *RARA* gene rearrangement and with the *RARG* gene rearrangement or dysregulation that presented abnormal promyelocytes fully in accordance with the APL phenotype.

## The Molecular Biology of Retinoic Acid Receptor γ (RARγ)

**Figure 1:**
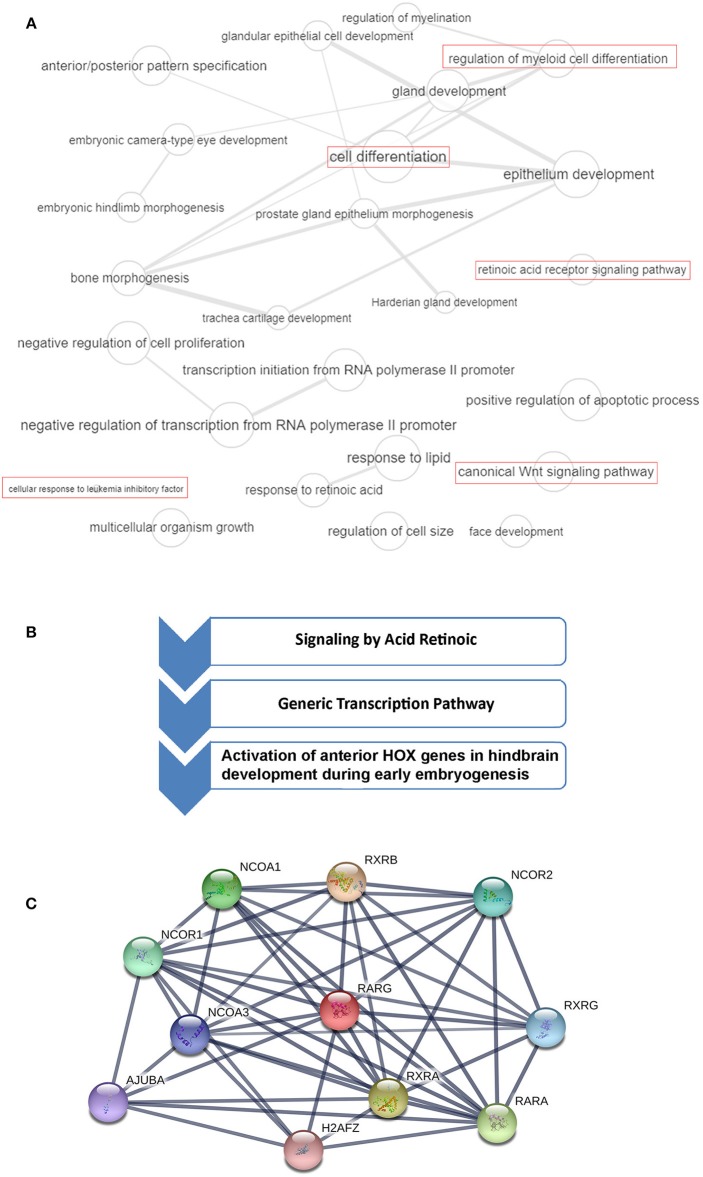
*RARG* gene ontology (GO), cellular pathways and protein-protein interaction analysis. **(A)** GO terms of *RARG* visualized by REVIGO (http://revigo.irb.hr/). Bubble size indicates the frequency of the GO term in the Gene Ontology Annotation database. Lines in the graph link highly similar GO terms, where their width indicates the degree of similarity. In red are highlighted RARG cellular processes that may be altered in leukemia. **(B)** *RARG* main cellular pathways derived from reactome database (https://reactome.org/). **(C)** RARG protein-protein interactions obtained by STRING database (https://string-db.org/). The interactions include direct (physical) and indirect (functional) associations.

RARγ belongs to the nuclear receptor superfamily and shares 90% homology with RARα and RARβ (Chambon, 1996). These three types of RARs have been discovered by cDNA cloning in both human and mouse and were identified as the only three members of the RAR family in mouse and man (Kastner et al., 1994). Moreover, all three RAR types have been characterized in the newt (Giguère et al., 1989; Ragsdale et al., 1989), RARβ cDNA has been cloned from chicken (Noji et al., 1991; Padanilam et al., 1991; Rowe et al., 1991; Smith and Eichele, 1991), RARα and RARγ cDNA have been found in Xenopus (Ellinger-Ziegelbauer and Dreyer, 1991; Blumberg et al., 1992) and have been isolated from zebrafish (Kastner et al., 1994), so these findings suggest that this RAR genes family exists in all vertebrates, mediating a series of effects, that may sometimes even be conflicting. RARγ is involved in several biological processes and cellular pathways, interacting with different proteins (Figure 1). The chromosomal mapping of *RARG* in humans is on 12q13, while *RARA* and *RARB* mapping is on 17q21.1 and 3p24, respectively (Mattei et al., 1988, 1991; Ishikawa et al., 1990). The protein structure of RARγ presents a DNA binding domain (DBD) that receives two “zinc fingers” that allow the protein to bind to specific DNA sequences in the promoter of their target genes, known as retinoic acid response elements (RAREs), and a ligand-binding domain (LBD) that confers the ligand binding specificity, assuming a ligand-inducible transactivation function (Kastner et al., 1990; Leroy et al., 1991; Zelent et al., 1991; Liu and Linney, 1993; Bastien and Rochette-Egly, 2004; Storlazzi et al., 2007; Al Tanoury et al., 2013). In detail, it is possible to recognize six different regions in the RAR primary sequence (A-F), which exhibit differential degrees of conservation (Kastner et al., 1994). The most conserved regions among the three RARs, within a given species, are the DBD (region C), the LBD (region E) and the region B which presents a promoter-specific transcription activation function (Nagpal et al., 1992). Although the D region, known as a hinge region, is well-conserved in its N-terminal portion, the central region appears to be more variable. Finally, the A and F regions differ notably among the three receptors (Kastner et al., 1994). For each RAR isotype it is possible to identify two or more isoforms; for regarding RARγ, two main isoforms (RARγ1 and RARγ2) have been identified, that show a different spatio-temporal expression. RARγ1 is found in the later phase of embryogenesis, as well as in the skin of newborns and adults, while RARγ2 is expressed in the early embryo (Kastner et al., 1990; Chambon, 1993). In the presence of a ligand, such as ATRA, RARs form heterodimers with the retinoid X receptors (RXRs) that operate as transcription factors, activating the RAREs regions in the target genes promoter (Mic et al., 2003; Bastien and Rochette-Egly, 2004). In detail, each RARs subtype presents a different sensitivity at different concentrations of ATRA: RARα activation occurs in the presence of high concentrations of ATRA, whereas RARγ activation requires the lowest amount of ligand (Beard et al., 2002).

## Investigation of RARγ Role

Although knockout mice studies have shown some functional redundancy among the different RAR isotypes, in reality each of them has a unique role in the processes of development and differentiation that cannot be replaced by the action of the other isotypes (Taneja et al., 1995; Mark et al., 2006). *RARG* null mice display growth deficiency (Lohnes et al., 1993); indeed, RARγ is highly expressed in the growth plate and its absence is associated with a reduced chondrocyte proliferation and decreased expression and deposition of proteoglycans (Williams et al., 2009). In addition, RARγ is required for the correct formation of the axial skeleton including anteriorization of the cervical and thoracic vertebrae (Lohnes et al., 1993; Wendling et al., 2001). Other studies have demonstrated that the genetic ablation of *RARG* in mice induced a decrease in bone mass due to an increase in osteoclastogenesis and consequent loss of trabecular bone mass (Walkley et al., 2007; Green et al., 2015). In addition, RARγ is crucial for the formation of normal alveoli and alveoli elastic fibers in the lung (McGowan et al., 2000) and its genetic ablation is associated with male sterility and with alterations of the prostatic glandular epithelia (Lohnes et al., 1993). Moreover, RARγ signaling is critical for epigenetic changes induced by retinoic acid (RA) (Kashyap et al., 2013). RARγ, as well as RARα, are crucial for hematopoietic development (Purton, 2007). Indeed, while RARα induces granulocytic differentiation, RARγ plays a central role in maintaining the balance between the self-renewal state of hematopoietic stem cells (HSCs) and their differentiation (Purton et al., 2006; Purton, 2007). Purton et al. reported that *RARG* null mice show reduced numbers of long-term repopulating HSCs and increased numbers of more committed hematopoietic progenitors, therefore *RARG*^−/−^ BM displayed significantly increased numbers of common myeloid progenitors and common lymphoid progenitors, unlike *RARG*^+/+^ BM. This finding suggests that loss of *RARG* induces an imbalance in HSC self-renewal decisions, supporting differentiation divisions. Moreover, Purton et al. have shown that RARγ signaling affected other genes known to elicit HSC self-renewal, such as *NOTCH1*, whose expression decreases severely in the absence of RARγ (Purton et al., 2006). Walkley et al., showed that *RARG* null mice exhibit a considerable increase in granulocytes in the peripheral blood (PB), in the BM and spleen, developing a myeloproliferative-like syndrome and displaying defects in both erythropoiesis and in B lymphopoiesis (Walkley et al., 2007; Dewamitta et al., 2014). Transplantation studies have confirmed that the expression of RARγ is essential in the BM microenvironment for a correct hematopoiesis to occur (Joseph et al., 2016). Indeed, Dewamitta et al. suggested that deficiencies observed in *RARG*^−/−^ mice are due to an aberrant *RARG*^−/−^ BM microenvironment, and therefore it is not an intrinsic cellular defect (Dewamitta et al., 2014). Green et al. also confirmed that RARγ is a crucial regulatory key for the presence of a healthy BM microenvironment, since mice with conditionally deleted *RARG* in more primitive limb bud-derived mesenchymal stem cells and their progeny, achieved through the use of *Prrx1-Cre*, showed alterations in the number of both osteoclasts and osteoblasts, with consequent modifications in the trabecular bone and an abnormal angiogenesis and B lymphopoiesis. These results seem to confirm the key role of RARγ in maintaining homeostasis in the various processes that occur in the BM microenvironment (Conserva et al., 2019), such as endochondral bone formation, angiogenesis, osteoclastogenesis and B lymphopoiesis (Green et al., 2018). Considering that vitamin A-derived retinoids play a central role in the growth and differentiation of a variety of cell types, RARγ in particular mediates various anti-proliferative and apoptotic effects of retinoids in certain tissues and cancer cells, such as melanoma and neuroblastoma cells (Spanjaard et al., 1997; Meister et al., 1998). Chen et al. also showed that the genetic ablation of *RARG* in a model of epidermal tumorigenesis enhanced the tumor incidence of Ras-transformed keratinocytes and was associated with retinoids resistance (Chen et al., 2004). Several studies, in recent years, have described various cases of patients carrying translocations involving *RARG* and showing a leukemic phenotype that resembles APL (Table 1). Therefore, it seems that *RARG*, just like *RARA*, can somehow rearrange and mediate oncogenic effects.

**Table 1:** AML with *RARG* rearrangements updated reported cases.

**No**.	**Age/gender**	**PB**	**Morphology**	**Cytogenetics**	**Molecular analysis**	**Therapy**	**ATRA responsivity**	**References**
1	35/M	WBC: 12 × 10^9^/L HB: 6.0 g/dL PLT: 8 × 10^9^/L	80% hypergranular promyelocytes with Auer rods	46, XY *t*(11;12)(p15;q13)[16] /46, XY[4]	*NUP98-RARG*	IA as induction therapy, chemotherapy followed by auto-HSCT as consolidation therapy	None	Such et al., 2011
2	45/F	WBC: 0.2 × 10^9^/L HB: 66g/L PLT: 60 × 10^9^/L	94.5% hypergranular promyelocytes with Auer rods	46, XX *t*(11;12)(p15;q13)[16]	*NUP98-RARG* *WT1* mutation	ATRA + ATO treatment, switched to IA as induction therapy	None	Luo et al., 2019
3	22/M	WBC: 112.6 × 10^9^/L HB: 82 g/L PLT: 92 × 10^9^/L	91.0% promyelocytes	46, XY *t*(11;12)(p15;q13)	*NUP98-RARG* *WT1* mutations	ATRA, ATO, +idarubicin, then HAA, DA as induction therapy	None	Zhang et al., 2019b
4	38/M	WBC: 1.68 × 10^9^/L HB: 8.0 g/dL PLT: 79 × 10^9^/L	65% promyelocytes	46, XY[20]	*CPSF6-RARG* *WT1* mutation	ATRA + RIF d1-25, MA	None	Qin et al., 2018
5	48/F	WBC: 0.81 × 10^9^/L HB: 4.2 g/dL PLT: 92 × 10^9^/L	89% hypergranular promyelocytes with Auer rods	92, XXXX[2]	*CPSF6-RARG* *DNMT3A* mutation	ATRA + ATO + IDA, IAG + ATRA + ATO, Decitabine as induction therapy	None	Liu et al., 2018
6	51/F	WBC: 20.15 × 10^9^/L HB: 6.5 g/dL PLT: 45 × 10^9^/L	87.5% hypergranular promyelocytes	46, XX, del(12)(p12)[2]/46, XX[18]	*CPSF6-RARG* *WT1, KRAS* mutation	ATRA + DNR, DA as induction therapy, HD-Ara-C followed by 3 + 7 regimens	None	Liu et al., 2018
7	26/M	WBC: 16.4 × 10^9^/L HB: 10.5 g/dL PLT: 120 × 10^9^/L	60% blasts and 15% promyelocytes	45, X, -Y [10]/45, idem, add(6)(q?13)[2]/46, XY[8]	*RARG-CPSF6* *BMPR1A, NEAT1, WT1* mutation	ATRA + IA as induction therapy	None	Miller et al., 2018
8	69/M	WBC: 1.5 × 10^9^/L HB: 123 g/L PLT: 204 × 10^9^/L	56% hypergranular promyelocytes	46, XY	*NPM1-RARG-NPM1*	ATO + ATRA treatment	None	Chen et al., 2019
9	64/F	WBC: 1.26 × 10^9^/L HB: 8.4 g/dL PLT: 120 × 10^9^/L	86.5% blasts and atypical hypergranular promyelocytes with Auer rods	46, XX,*t*(12;15)(q13;q22)	*PML-RARG*	IA as induction therapy, HD-Ara-C, followed by allo-HSCT as consolidation therapy	NA	Ha et al., 2017
10	42/M	WBC: 18,500/μL HB: 8.6 g/dL PLT: 118,000/μL	83% blast cells; among them 30% with hypergranulated cytoplasm with Auer rods and PCH anomaly	46, XY[20] der(2) chr. (17q21.32-q25.3 duplication and 2q35-37.3 deletion)	*EZH2* mutation *RARA* and *RARG* downregulation	3 + 7 regimens, then FLAG-IDA	NA	Coccaro et al., 2018
11	33/F	WBC: 35.6 × 10^9^/L HB: 76 g/L PLT: 67 × 10^9^/L	4.0% myeloblasts and 70.5% aberrant promyelocytes	46, XX	*ELL-MLL* *RARG* upregulation *RARA* downregulation	ATO treatment	NA	Zhang et al., 2019a

*No., number; PB, peripheral blood; ATRA, all-trans retinoic acid; Ref., reference; M, male; F, female; WBC, white blood cell; HB, hemoglobin; PLT, platelet; IA, Idarubicin and cytarabine; Auto-HSCT, autologous hematopoietic stem cell transplantation; ATO, arsenic trioxide; HAA, homoharringtonine, clacinomycin, and cytarabine; DA, Daunorubicin and cytarabine; RIF, Realgar-Indigo naturalis formula; MA, mitoxantrone and cytarabine; IDA, Idarubicin; IAG, Idarubicin, cytosine arabinoside, and G-CSF; DNR, Daunorubicin; HD-Ara-C, High-dose cytarabine; Allo-HSCT, allogeneic hematopoietic stem cell transplantation; FLAG-IDA, Fludarabine, Cytarabine, G-CSF, Idarubicin; NA, not available*.

## *RARG* in Myeloid Malignancies

**Figure 2:**
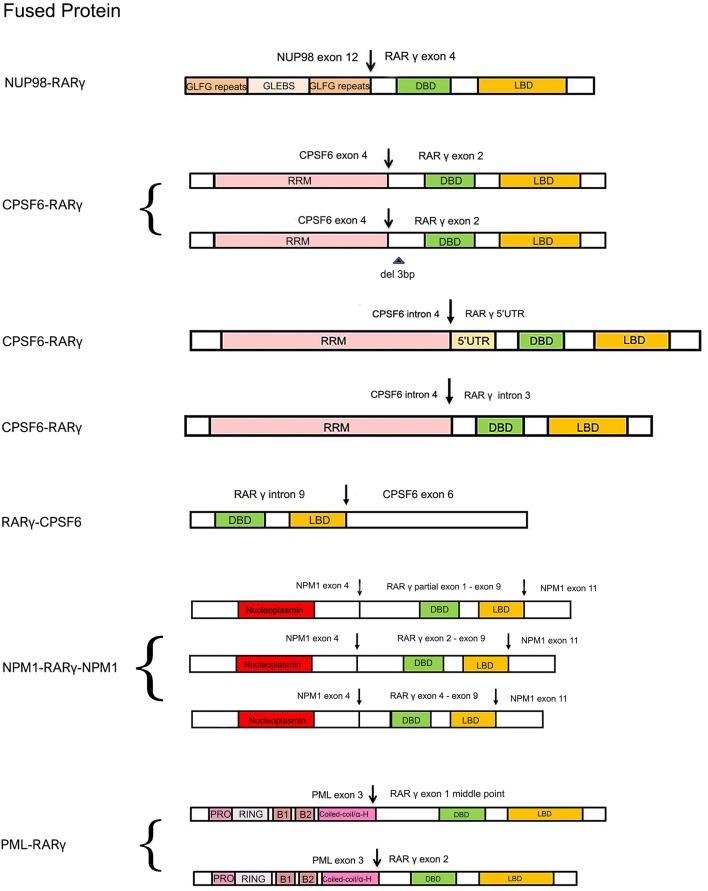
Schematic comparison of RARγ fusion proteins identified or expected in AML reported cases. The variably colored rectangles indicate all the important domains of RARγ and its partner proteins. The arrows indicate DNA break points. Curly brackets are used to group fusion proteins all found in one patient. GLFG, Gly-Leu-Phe-Gly; GLEBS, GLE2p-binding sequence; RRM, RNA recognition motif; PRO, proline-rich region; RING, RING finger; B1/B2, B-Box; Coiled-coil/α-H, coiled-coil/alpha-helical; NLS, nuclear localization signal.

*RARG* rearranged leukemia is a rare specific subtype of AML, but its characterization is very ambiguous because its features are so strongly similar to APL. Indeed, patients presenting *RARG* rearrangements share typical APL clinical presentations, coagulation abnormalities and morphological and immunophenotypic features of BM (Luo et al., 2019). *RARA* rearrangements are known to generate APL, and rearrangements involving *RARB* or *RARG* can resemble APL. Typical APL is characterized by recurrent *PML-RARA* expression, but up to now, to our knowledge, eleven cases with *RARG* dysregulation have been identified (Such et al., 2011; Ha et al., 2017; Liu et al., 2018; Miller et al., 2018; Qin et al., 2018; Chen et al., 2019; Luo et al., 2019; Zhang et al., 2019b) (Figure 2). As previously reported by Conserva et al., the three RARs are highly homologous so it is not unexpected that rearrangements involving both *RARB* and *RARG* can generate similar diseases (Conserva et al., 2019). However, the involvement of *RARG* principal cellular pathways in AML needs to be further clarified (Figure 1A).

### NUP98-RARG

The first AML patient with a *RARG* rearrangement, *NUP98-RARG*, was reported in 2011 (Such et al., 2011). *NUP98* is the nucleoporin 98 gene mapped on chromosome 11p15, frequently involved in several rearrangements in both myeloid and lymphoid malignancies (Romana et al., 2006; La Starza et al., 2009). The patient (no. 1, Table 1) was a 35-years-old man exhibiting morphologic and clinical characteristics resembling APL. The genomic aberration found was translocation *t*(11;12)(p15;q13), plus a 1.0 Mb microdeletion in 11p15 and a 2.5 Mb microdeletion in 12q13. *NUP98* exon 12 was fused in frame to *RARG* exon 4. Interestingly, the *NUP98* 5′ region encodes Gly-Leu-Phe-Gly-repeats (GLFG) and GLE2p-binding sequence (GLEBS)-like motifs, which act as transcription activators, providing docking sites for the nuclear transport of RNA and protein between the nucleus and the cytoplasm; while the 3′ region *RARG* involved in the fusion includes DBD and LBD. The mechanism underlying the transformation mediated by *NUP98-RARG* is unknown. The patient was started on ATRA treatment that was soon discontinued and switched to a standard 7 + 3 schedule (cytarabine and idarubicin) as induction therapy, then he achieved complete remission (CR). Two years later, the patient relapsed; he achieved second CR but then he died due to an infection complication (Such et al., 2011). Several studies were conducted by Qiu et al. focused on the RARγ involvement in retinoid signaling. Using green fluorescent protein-tagged versions of these proteins and analyzing their signal, they demonstrated that the novel NUP98-RARγ fusion protein acquires a different subcellular localization compared to NUP98 and wild-type RARγ. This localization is strongly dependent on the RARγ DBD domain, as shown by different experiments conducted using mutant variants of NUP98-RARγ. They also verified, through co-immunoprecipitation assays, the NUP98-RARγ ability to form aberrant homo-dimers and homo-oligomers, but this feature is granted by the NUP98 portion of the chimeric protein. The RARγ fraction is essential for interacting with RXRα: they also demonstrated that the RXR agonist can suppress the NUP98-RARγ/RXR connection. Luciferase reporter experimental results also suggested that NUP98-RARγ may have equivalent transcriptional properties to those of the RARα fusion protein, involved in APL, on its downstream targets. The oncogenic potential of NUP98-RARγ was confirmed by retroviral transduction/transformation assay in murine HSCs; furthermore, the cellular transformation ability requires both RARγ and NUP98 contributions and these transformed cells are sensitive to RXR agonist and ATRA treatment (Qiu et al., 2015). It was necessary to conduct several experiments to test ATRA responsivity since the first patient with *RARG* rearrangement received ATRA simultaneously to chemotherapy, so it was difficult to evaluate the effective *in vivo* responsivity to ATRA, even if it has been reported that fusion proteins generated with RARγ confer responsiveness to ATRA *in vitro* and *in vivo* (Marinelli et al., 2007, 2009). The *in vitro* studies showed that the *NUP98/RARG* rearrangement confers ATRA resistance (Such et al., 2014). Moreover, another patient (no. 2, Table 1) with *NUP98/RARG* exhibited a primary resistance to ATRA treatment (Luo et al., 2019). Sequencing of the fusion transcript showed also that *NUP98* exon 12 was fused in frame to *RARG* exon 4, in the first patient described, and the Wilms' tumor (*WT1*) mutation was also identified. These studies point out an evident paradox of ATRA resistance/responsiveness *in vitro* and *in vivo*: it may depend on the culture assay condition or on the acquisition of additional mutations causing resistance (Such et al., 2014; Qiu et al., 2015). A third case of *NUP98-RARG* AML (no. 3, Table 1) was observed by Zhang et al. The break points were the same as in the other two cases reported in literature. In addition, two *WT1* mutations were found. Given the two previous cases, the author established that *NUP98-RARG* AML is sensitive to the standard 3+7 regimen, and that allogeneic HSC transplantation (allo-HSCT) may be the best procedure to achieve CR. It is possible that additional genetic mutations can cause ATRA resistance (Zhang et al., 2019b).

### *CPSF6-RARG* and *RARG-CPSF6*

Cleavage and polyadenylation specific factor 6 (CPSF6) is a subunit of cleavage factor I, which is an RNA binding protein complex involved in the alternative cleavage and polyadenylation process (Millevoi and Vagner, 2010). Although several studies have demonstrated the involvement of CPSF6 in HIV infection and breast cancer (Rasheedi et al., 2016; Binothman et al., 2017), the role of CPSF6 in myeloid leukemia has not yet been clarified. The first case of *CPSF6-RARG* rearrangement was found by Qin et al. in an AML 38-years-old male (no. 4, Table 1) presenting clinical and morphological features of classic APL. Both *CPSF6* and *RARG* map on chromosome 12. In addition, a *WT1* mutation was identified. The rearrangement within chromosome 12 led to the generation of two different fusion transcripts, depending on the breaking points: the major transcript made up by *CPSF6* exon 4 fused to *RARG* exon 2 and the minor transcript by a deletion of 3 bp at the 5′ end of *RARG* exon 2. As regards therapy, the patient was treated with ATRA, but the ATRA treatment was interrupted because of the differentiation syndrome, so the best therapy has still to be established (Qin et al., 2018). Two cases of *CPSF6-RARG* rearrangements were then identified, with different genomic aberrations. In patient no. 5 (Table 1) a tetraploidy karyotype in two metaphases and a *DNMT3A* mutation were found; in patient no. 6 (Table 1) a del(12)(p12) and *WT1* and *K-RAS* mutations were detected. Both patients showed ATRA resistance. The breakpoint in *CPSF6* was located in intron 4 in both patients, while there were 2 breakpoints in intron 3 or 5′UTR and the telomeric region of exon 9 of *RARG*. The 3′ region of *RARG* (from exon 1 or exon 4 to exon 9) was reversed and fused in frame with the 5′ region of the *CPSF6* gene (from exon 1 to exon 4). Even in these rearrangements, RARγ retained DBD and LBD domain, as well as in the *NUP98-RARG* rearrangements described before. Moreover, the *RARG* breakpoint in patient no. 5 is the same as in *NUP98-RARG*, patient no.1 (Table 1) (Such et al., 2011; Liu et al., 2018). These two cases were included in a study which enrolled 1,401 patients with suspected AML. Among these, 19 patients had alternative *RARA* or *RARG* rearrangements. The paper shows an interesting point about the prognostic impact of the presence of this kind of rearrangements: the overall survival and the leukemia-free survival were significantly lower in patients with alternative *RARA* or *RARG* fusions in comparison with patients with the *PML-RARA* classic gene fusion (Wen et al., 2019). Interestingly, the *RARG-CPSF6* fusion was identified in a 26-years-old patient (no. 7, Table 1) with AML resembling APL, with a profoundly rearranged region on chromosome 12, where both *RARG* and *CPSF6* are mapped, featuring breakpoints in these two genes and in the eukaryotic translation initiation factor 4B (*EIF4B*). *RARG* intron 9 was fused to *EIF4B* intron 8, but there was a deletion after this point and *EIFB4* was then fused into the intron leading into *CPSF6* exon 6. These rearrangements led to the formation of a novel *RARG-CPSF6* transcript fusion, but there was no evidence of the corresponding protein (Miller et al., 2018).

### NPM1-RARG-NPM1

Recently, another gene involved in the *RARG* rearrangement was identified. *NPM1* is the nucleophosmin 1 gene mapped on chromosome 5 and it has been indicated as a partner of *RARA* in APL variant translocations (Kikuma et al., 2015). In this AML case (no. 8, Table 1), *NPM1* is fused with *RARG*. There was a deletion of about 16 kb of *NPM1* from intron 4 to intron 10 and an insertion of about 23 kb of *RARG* from 5′ UTR to intron 9. Sequencing analysis revealed that both *NMP1* and *RARG* had two breakpoints each. The *NPM1* breakpoints were located in intron 4 and intron 10; the *RARG* breakpoints were identified in 5′UTR and intron 9. The presence of three *NPM1-RARG-NPM1* different chimeric transcripts was confirmed: from exon 1 to exon 4 of *NPM1* fused with *RARG*, from partial exon 1 to exon 9, then fused with exon 11 of *NPM1*; exon 1 to exon 4 of *NPM1* fused with *RARG*, from exon 2 to exon 9, then fused with exon 11 of *NPM1*; exon 1 to exon 4 of *NPM1* fused with *RARG*, from exon 4 to exon 9, then fused with exon 11 of *NPM1*. In all three transcripts *RARG* maintained its DBD, while the deletion of *RARG* exon 10 resulted in the loss of LBD 25 amino acids. The patient, like others harboring *RARG* rearrangements, had an APL-like clinical presentation and morphological features, so he received ATRA treatment, but resulted resistant. This is a unique case of AML with NPM1*-RARG-NMP1* chimeric fusion (Chen et al., 2019).

### PML-RARG

*PML* is well-known to be involved in translocation with *RARA* in APL, in which they produce the PML-RARα chimeric protein that behaves as an altered RARα, and represses the transcription of RA target genes (Kakizuka et al., 1991). *In vitro* and *in vivo* studies had already demonstrated that PML-RARγ has an oncogenic potential (Marinelli et al., 2007, 2009). Ha et al. identified a novel involvement of *PML* in a fusion with *RARG* in an AML patient (no. 9, Table 1) with morphologic and immunophenotypic features of classical APL. The translocation *t*(12;15)(q13;q22) was identified; the breakpoints were on intron 3 of *PML* and the 5′ upstream region of *RARG*. Two kinds of *PML-RARG* transcripts arise from this gene fusion: a longer transcript (*PML* exon3-*RARG* exon1 middle point) and a shorter one (*PML* exon3-*RARG* exon2). RARγ preserves its DBD and LBD, as in the other cases. It may be interesting to note that the fusion site of *PML* in this rearrangement is consistent with breakpoint cluster region 3 (bcr3) type of *PML-RARA*. The patient had intermittent ATRA treatment, so the sensitivity was not clarified (Ha et al., 2017).

### *RARG* Epigenetic Alteration

Physiologically, the enhancer of zeste 2 polycomb repressive complex 2 subunit *(EZH2)* is involved in gene silencing thanks to its capability to recruit DNA methyltransferases (DNMTs) for gene repression and its interaction with two epigenetic repression systems (Viré et al., 2006; Sashida and Iwama, 2017). Epigenetic alterations of *RARs* genes can also lead to an AML resembling APL. In one patient (no. 10, Table 1) presenting similar features of APL an *EZH2* mutation was found by our group, without any *RARs* rearrangement (Coccaro et al., 2018). The *EZH2* mutation was in its DNMT binding region. Analysis showed a profound downregulation of *RARA* and *RARG* expression compared with APL, AML and normal control pools (Coccaro et al., 2018). Zhang et al. also reported that a patient harboring *ELL-MLL* (no. 11, Table 1) can seem to have APL; in particular *RARG* was upregulated (Zhang et al., 2019a).

## Conclusions

Despite the diversity of gene partners, *RARG* rearrangements identified up to now have shown similar features, such as the retention of DBD and LBD RARγ domains; the presence of the chimeric transcript, but not always of the protein, and ATRA resistance. Probably, DBD/LBD maintenance affects the fusion protein different nuclear localization and is involved in the leukemogenesis process, leading to an alteration especially of the granulocyte lineage, as well as *PML-RARA*. Nevertheless, RARγ, like RARα, is critical for the physiological hemopoietic process, although its role is not comparable. In fact, RARγ is involved in maintaining a balance between the self-renewal and differentiation of HSCs rather than in the granulocytic differentiation. Surely, RARγ exhibits an indirect role in this, but it is not clear why *RARG* rearrangements are capable of resembling APL phenotype. Hence, this aspect must be more closely investigated, mainly because these patients seem to be ATRA-resistant. Indeed, ATRA responsivity also needs to be clarified since *in vitro* and *in vivo* studies have shown contrasting results. In addition, in most reported cases, several mutations in different genes have been identified, especially in the *WT1* gene. This factor could be crucial in the oncogenic process and might also influence ATRA sensitivity. In our opinion, further studies are needed to better understand the oncogenic role of *RARG* rearrangements, and the molecular features of both the chimeric transcript and fusion protein, in particular to define the best therapy for these APL atypical patients.

## Author Contributions

MC and IR conceived and wrote the review. LA and AZ performed the literature analysis. FA and GS supervised and approved the final manuscript.

### Conflict of Interest

The authors declare that the research was conducted in the absence of any commercial or financial relationships that could be construed as a potential conflict of interest.

## Abbreviations

RARγ: retinoic acid receptor γ
RARα: retinoic acid receptor α
RARβ: retinoic acid receptor β
APL: acute promyelocytic leukemia
BM: bone marrow
AML: acute myeloid leukemia
ATRA: all-trans retinoic acid
ATO: arsenic trioxide
PML: promyelocytic leukemia protein
DBD: DNA binding domain
RAREs: retinoic acid response elements
RXRs: retinoid X receptors
RA: retinoic acid
HSCs: hematopoietic stem cells
PB: peripheral blood
NUP98: nucleoporin 98
CR: complete remission
WT1: Wilms’ tumor
Allo-HSCT: allogeneic HSC transplantation
CPSF6: Cleavage and polyadenylation specific factor 6
OS: overall survival
LFS: leukemia free survival
EIF4B: eukaryotic translation initiation factor 4B
NPM1: nucleophosmin 1
bcr3: breakpoint cluster region 3
EZH2: enhancer of zeste 2 polycomb repressive complex 2 subunit
DNMTs: DNA methyltransferases.
